# A review of the use of health examination data from the Health Survey for England in government policy development and implementation

**DOI:** 10.1186/2049-3258-72-24

**Published:** 2014-07-15

**Authors:** Oyinlola Oyebode, Jennifer S Mindell

**Affiliations:** 1 Department of Epidemiology and Public Health, UCL (University College London), London, UK

**Keywords:** Policy, Evidence, Surveillance, Health examination survey

## Abstract

**Background:**

Information is needed at all stages of the policy making process. The Health Survey for England (HSE) is an annual cross-sectional health examination survey of the non-institutionalised general population in England. It was originally set up to inform national policy making and monitoring by the Department of Health. This paper examines how the nurse collected physical and biological measurement data from the HSE have been essential or useful for identification of a health issue amenable to policy intervention; initiation, development or implementation of a strategy; choice and monitoring of targets; or assessment and evaluation of policies.

**Methods:**

Specific examples of use of HSE data were identified through interviews with senior members of staff at the Department of Health and the Health and Social Care Information Centre. Policy documents mentioned by interviewees were retrieved for review, and reference lists of associated policy documents checked. Systematic searches of Chief Medical Officer Reports, Government ‘Command Papers’, and clinical guidance documents were also undertaken.

**Results:**

HSE examination data have been used at all stages of the policy making process. Data have been used to identify an issue amenable to policy-intervention (e.g. quantifying prevalence of undiagnosed chronic kidney disease), in strategy development (in models to inform chronic respiratory disease policy), for target setting and monitoring (the 1992 blood pressure target) and in evaluation of health policy (the effect of the smoking ban on second hand smoke exposure).

**Conclusions:**

A health examination survey is a useful part of a national health information system.

## Background

Reliable health data and statistics are necessary to underpin health policies, strategies, and their evaluation and monitoring, as well as providing the basis for sound health information for the general public. Health surveys that recruit a representative sample of the population are the most appropriate way of collecting data on health determinants, morbidity, and unmet health need for use by national policy-makers. Health interview surveys collect data through interview or self-administered questionnaires, while health examination surveys involve taking some physical and/or biological measurements to complement self-reported data.

The National Health Survey undertaken in the USA in 1935 and 1936 was the first large survey examining health status. Its aim was to study the extent and nature of disability in the general population, particularly chronic disease and physical impairment [1]. It became the main data source contributing to the government’s health proposals [1]. Since then the National Health and Nutrition Examination Survey (NHANES) has run intermittently since the 1960s and on a rolling basis since 1999; anecdotal evidence links this survey to many health policy decisions [2].

Seven European countries as well as South Korea, Japan, Mexico and Australia, have conducted more than one health examination survey [3,4]. Finland has the longest running health examination survey in Europe, conducted every 5 years since 1972. Other health examination surveys have been conducted around the world; however most of these have happened just once. Some countries are planning their first health examination survey and others are seeking justification to fund one [5].

The Health Survey for England (HSE) is an annual health examination survey which started in England in 1991 [6] and resulted from a 1988 ‘Command paper’ , *Public Health in England*[7]. (In the UK, command papers are reports presented to parliament, as a statement of government policy on a specific subject). At the time the HSE was set up, several sources reported a lack of morbidity data needed to measure and monitor the health of the UK population [8,9].

The HSE samples a nationally representative random cross-section of the free-living general population of England. Following an advance letter to the selected households, an interviewer visits to recruit up to ten adults and up to two children per household. The interviewer collects socio-economic data and information on health and health-related behaviours, and measures height and weight. Participants who agree are then visited by an experienced, trained nurse who takes physical measurements, such as waist and hip circumferences and blood pressure, and collects biological samples (i.e. blood, urine and/or saliva) and information on medication use. Field-staff undergo training, with refresher training annually. Data on adults has been collected yearly since 1991 and on children from 1995 onwards. In addition to an annual published report, data are available through the UK Data Service and are provided to the Department (Ministry) of Health directly [6].

Many measurements and samples have been taken by the HSE nurses since 1991 (Table 1). These measurements are essential to identify public health relevant health indicators where other survey methods, such as self-report, may not be reliable for prevalence estimation [10-13]. In order to select measures and samples included in the survey each year, each individual policy team within the Department of Health considers what its priorities and information needs are. If questions and/or measurements from the HSE would be particularly useful for up-coming policy development, they put a proposal to Health and Social Care Information Centre (HSCIC) which is discussed and costed. Where a policy team can fund this from their budget and receives approval from senior finance officers, the information is collected.

**Table 1:** Measurements and samples taken as part of the HSE, by year

	**1993**	**1994**	**1995**	**1996**	**1997**	**1998**	**1999**	**2000**	**2001**	**2002**	**2003**	**2004**	**2005**	**2006**	**2007**	**2008**	**2009**	**2010**	**2011**	**2012**
***Measurements***																				
Blood pressure 5+	•	•	•	•	•	•	•^3^	•	•	•	•	•	•	•	•	•	•^8^	•	•	•
Demi span		•	•	•		•	•^3^	•^2^	•				•^2^	•^2^	•^2^	•^6^				
ECG							•^3^	•^1^												
Grip strength													•^2^							
Physical function – balance													•^2^							
Lung function 7+			•	•	•		•^3^		•	•		•						•		
Infant length									•	•	•	•	•	•	•					
Step test																•^7^				
Upper arm circumference			•	•	•	•	•^3^													
Waist/hip circumference 11+	•	•			•	•	•^3^	•^2^	•	•	•	•	•	•	•	•	•	•	•	•
Walking speed													•^2^							
***Blood sample***																				
C reactive protein						•	•^3^				•	•		•			•			
Cotinine	•	•		•	•															
Creatinine																	•	•		
Fibrinogen	•	•				•	•^3^	•^1^			•	•	•^2^	•			•			
Influenza antibodies																				•
Gamma gt	•	•																		
Glycated haemoglobin	•	•					•^3^	•^1^			•	•	•^2^	•		•	•	•	•	•
Haemoglobin + ferritin	•	•	•	•	•	•	•^3^	•^1^	•	•		•	•^2^	•			•			
HDL cholesterol						•	•^3^	•^1^			•	•	•^2^	•		•	•	•	•	•
IgE/HDM Ige			•	•	•		•^3^		•	•		•								
MCV								•^1^					•^2^							
Serum albumin								•^2^					•^2^							
Serum transferin													•^2^							
Total cholesterol	•	•				•	•^3^	•^1^			•	•	•^2^			•	•	•	•	•
Vitamin D								•^2^					•^2^					•		
Vitamin B12													•^2^							
***Fasting blood sample***																				
Glucose							•^3^				•	•								
LDL cholesterol							•^3^				•	•								
Triglycerides							•^3^				•	•								
***Saliva sample***																				
Cotinine				•	•	•	•^3^		•^4^	•^4^	•^4^	•^4^	•^4^	•^4^	•^5^	•^5^	•^5^	•^5^	•^5^	•^4^
***Urine sample 16+***																				
Sodium, potassium, creatinine											•	•	•	•	•		•	•		•
Albumin																	•	•		
Melatonin																		•		

^1^65+ in care homes only; ^2^All 65+; ^3^Minority ethnic group respondents only; ^4^Children aged 4–15; ^5^Children aged 4–15, adults 16+; ^6^Aged 25–44, 65+; ^7^Aged 16–74; ^8^Aged 16+.

There are many stages of policy making: from identifying, quantifying and promoting recognition of health issues to seek or to justify policy intervention; through strategy development, including economic analysis, impact assessment, selection of targets; to target monitoring and policy evaluation. This paper reviews use of HSE examination data collected at the nurse visit, at each policy stage. Specific examples are presented. There is no previously published analysis of the use of health examination data for policy making and monitoring, however this work complements our examination of the use of measured heights and weights in obesity policy, presented elsewhere [10].

## Methods

Specific examples of use of HSE data were identified through interviews with 18 senior members of staff at the Department of Health and at the HSCIC, both having responsibilities for England. Three of these interviewees were former staff from the Department of Health. Initial key informants were purposively sampled for their experience of working with HSE data; ‘snowball’ sampling strategies were used to reach additional staff. Interviews took place between May and July 2012.

Policy documents referred to by interviewees were retrieved for review, and reference lists of associated policy documents were checked. Systematic searches of Chief Medical Office reports, command papers, and clinical guidance documents were also undertaken. These documents were read, looking for use of HSE data in the text, tables or figures, or for the HSE in the reference list. A table was compiled (Additional file 1: Table S1) and was shown to interviewees to enable them to comment on whether any important examples had been forgotten. Specific examples are presented below, all other uses identified are outlined in Additional file 1: Table S1.

## Results

### Identifying an issue amenable to policy intervention

Chronic Kidney Disease (CKD) is associated with serious consequences including end-stage renal disease and cardiovascular diseases. By identifying CKD, it is possible to reduce the risk of these outcomes through preventative treatment [14]. Measurement of the national kidney disease burden is necessary to understand disease trends and aid the development of evidence-based policy [14].

In 2009 and 2010, serum creatinine and urinary albumin were collected as part of the HSE and used to calculate survey participants’ estimated glomerular filtration rates (eGFR) and the presence of albuminuria, respectively. These clinical markers of CKD were used to derive the overall prevalence of CKD in England, as well as prevalence by age, sex and socioeconomic status.

Before HSE data existed, prevalence of CKD in England was estimated using serum creatinine measures recorded on primary care computer systems, first as examined by researchers [15], and since 2006, through the Quality Management Analysis System (QMAS), a system that supports the calculation of practice-based performance payments to primary care practices (general practitioners, GPs) in England. QMAS reported that the prevalence of CKD in England was 4.3% in 2009–10 [16].

Relying on primary care data is not ideal for several reasons. First, it is known that GP registers overstate the true number of registered patients (known as GP list inflation), for example because of the delay in updating lists when patients move to a different practice or die [17]. This increases the denominator, thereby decreasing the apparent prevalence. Secondly, there are people living in England who are not registered with any GP; if these people have CKD, they would be missing from the numerator as well as the denominator. Thirdly, there may be under-recording by GP practices of diagnosed CKD in their patients due to time constraints or IT failure. Finally, and most importantly, there are patients with CKD who are registered with a GP but who have not been tested for, and therefore do not have a diagnosis of, CKD.

Based on eGFR data from the HSE 2009 and 2010, it is estimated that 6% of men and 7% of women have stage 3–5 CKD [11]. The difference between these figures and those from QMAS suggests that one-third of people with CKD in England are not represented in the QMAS figures and this could mean that a third of people with CKD are not diagnosed.

Further to this, each year that CKD prevalence has been reported through QMAS, prevalence has increased - from 3.0% in 2006–07, to 3.7% in 2007–08, 4.1% in 2008–09 and 4.3% in 2009–10. This is evidence that CKD ascertainment is increasing in GP surgeries, but does not distinguish between an increase in prevalence and an increase in diagnosis. The kidney team at the Department of Health were able to commit funding to examine stored blood samples collected from HSE participants in 2003–05 to produce trend data for the population prevalence of CKD from 2003–05 to 2009–10. Department of Health staff stated that whether the trend is going up or down, this information will feed into future policy work.

The HSE CKD prevalence data have influenced National Institute for Health and Care Excellence (NICE) guidance on CKD, including quality standards and commissioning of services, as well as being cited amongst reasons for reviewing clinical guidance [18-20]. They have been used in calculation of the financial costs of CKD, producing figures that help urge policy action to prevent CKD [21,22]. The HSE data have also been used to model prevalence at the local level to drive local action on CKD [23].

These examples demonstrate how accurate prevalence data, collected through the HSE, has been used to advocate for CKD at a policy level. Other specific examples of using HSE examination data to identify, quantify or highlight an issue amenable to policy intervention are shown in Additional file 1: Table S1. These include insight into the prevalence of undiagnosed hypertension, Chronic Obstructive Pulmonary Disease (COPD) and diabetes; examination of the prevalence and risk factors for vitamin D deficiency, iron deficiency anaemia, physical inactivity, raised dust-mite specific IgE and raised cholesterol; and presentation of cotinine data to support policy action on second-hand tobacco smoke.

### Strategy development

As with the CKD example above, clinical markers for COPD, specifically lung function tests, have been examined in HSE participants in eight of the past 20 years, allowing population prevalence to be examined and compared with the prevalence of diagnosed COPD in primary care data. HSE 2001 data have been used to model estimated COPD prevalence in the UK [24-26]. Shahab et al’s study modelled the HSE 2001 spirometry data categorised using the joint American Thoracic Society (ATS) and European Respiratory Society (ERS) guidelines. Their results were used by the British Lung Foundation to estimate that there were 3.7million cases of COPD in the UK [27]. However, primary care data showed only 900,000 diagnosed cases at that time. The British Lung Foundation therefore launched a campaign to find the ‘missing millions’ – the 2.8 million people in Britain with undiagnosed COPD - so that they could receive treatment (both medication and lifestyle advice, particularly support for smoking cessation) to reduce the individual and national burden of morbidity, disability, and premature mortality [27].

HSE data were also used in the development of the Department of Health strategy for COPD. *The Outcomes Strategy for COPD and Asthma* examined the prevalence of undiagnosed COPD and the ratio of diagnosed to expected prevalence of COPD by geographical area, citing studies which used HSE lung function data [28]. HSE COPD data were used in models that were used to fulfil the UK legal requirement to consider the effect of all government strategy on nine ‘protected characteristics’: age, disability, gender reassignment, marriage and civil partnership, pregnancy and maternity, race, religion or belief, sex, and sexual orientation. Interviewees told us that COPD data were further used in strategy implementation, making the case for the COPD outcomes strategy with healthcare professionals, and encouraging their acceptance of its recommendations.

Lastly, clinical guidance development has also used the COPD data collected by the HSE. A cost-effectiveness model comparing treatment with combinations of long-acting-beta-agonists, long-acting muscarinic antagonist and inhaled corticosteroids in people with severe/very severe COPD used Health Survey for England data to set up the estimated distribution of severity stages in people diagnosed with COPD in England [29].

Specific ways that HSE examination data have been used in strategy development in other disease areas are shown in Additional file 1: Table S1. These include the use of hypertension, glycated haemoglobin and cholesterol data in the economic modelling for vascular checks; and urinary albumin and creatinine data supporting the calculation of the human and economic costs of CKD.

### Target setting and monitoring

Like the HSE, the *Health of the Nation* strategy resulted from recommendations made in the 1988 command paper, *Public Health in England*. The *Health of the Nation* 1992 was the first attempt by a UK government to take a strategic view of health [30]. A range of targets to measure improvement in the population’s health were set in the *Health of the Nation*; the newly commissioned HSE was one way in which targets would be monitored, where existing health information was lacking. One such information need was data on which to base a blood pressure target. For this reason, the 1992 blood pressure target stated *“baseline to be derived from new national health survey”.* Once this data had been collected, the target became *“To reduce mean systolic blood pressure in the adult population by at least 5mm Hg by 2005”.* Monitoring of this target was to be done using HSE data [31].

**Figure 1:**
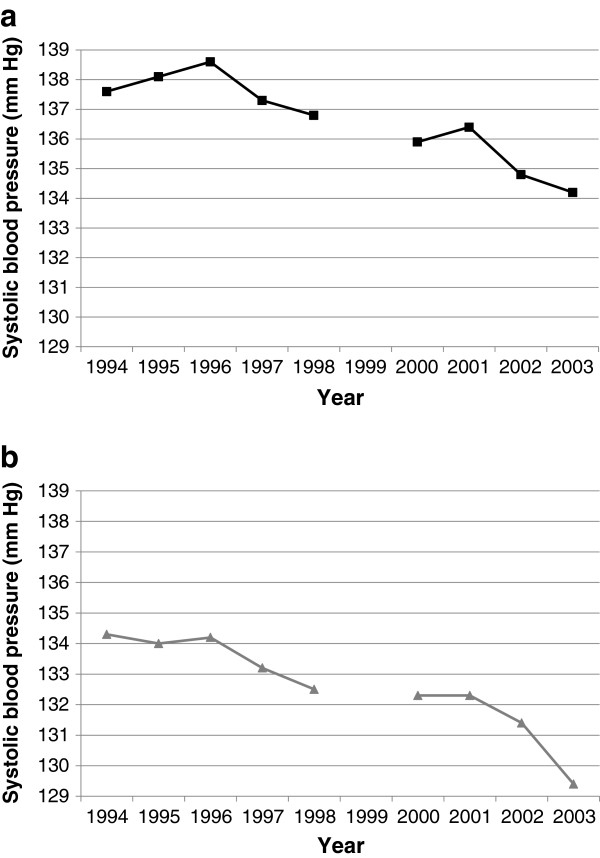
**Changes in systolic blood pressure over time.** Health of the Nation target: To reduce mean systolic blood pressure in the adult population by at least 5 mmHg by 2005 from the 1994 baseline. **a)** Men **b)** Women.

When this target was set, the detection and management of hypertension was typically characterised by the ‘rule of halves’: 50% of cases have been diagnosed, of which 50% are treated, and 50% of those are controlled [32]. A number of government policies were introduced in England to improve this, including the Quality and Outcomes Framework [33] for GPs introduced in 2004, which provided financial incentives to measure blood pressure and treat hypertension adequately [33]. Studies using HSE data from 1998, 2003, and 2006 showed that awareness of hypertension rose in men between 1998 (40% of those with ‘survey-defined hypertension’ (i.e. a systolic blood pressure of ≥ 140 mmHg, a diastolic blood pressure of ≥ 90 mmHg, or taking medication for hypertension)) and 2003 (60%) but changed little thereafter (62% in 2006), while in women it rose from 58% in 1998 to 64% in 2003 and to 71% in 2006. The proportion on treatment had risen in both sexes, from 33% in 1998 to 54% in 2006 in men and 44% to 62% respectively in women. Control of blood pressure levels had also risen, from 13% to 26% of men and 16% to 32% in women with hypertension over these eight years; among those reporting antihypertensive medication, it rose from 48% in 2003 to 52% in 2006 in men and from 44% to 53% respectively in women [34,35]. Further improvements were apparent by 2011, with 63% of men and 61% of women on antihypertensive treatment having controlled blood pressure [12].Assessment of population systolic blood pressure, using measurements undertaken by the HSE nurses, shows that this target was achieved ahead of time (Figure 1).

Other targets set and/or monitored using HSE examination data are shown in Additional file 1: Table S1. The hypertension data has been used to monitor inequality, through an indicator developed by the WHO. Cotinine measures from children have been used to monitor the proportion of homes where both parents are smokers but where the home itself has been declared smoke free.

### Evaluation and review of policies

Passive smoking has been assessed in the HSE through cotinine, in blood in earlier years and in saliva since the mid-1990s. The smoke free law, which banned smoking in work and public places, was passed as part of the 2006 Health Bill and came into force on 1^st^ July 2007 in England. The primary aim was to decrease the exposure of non-smoking adults to tobacco smoke, particularly those working in the hospitality industry, where clients’ smoke was responsible for high levels of environmental tobacco smoke.

Data from salivary cotinine in non-smoking adults, collected by the Health Survey, demonstrates that these intended consequences of the smoking ban occurred as expected [36,37], with a marked decrease in mean salivary cotinine in non-smoking adults and an increase in the number with no detectable cotinine. This provides some evidence of policy success.

However, prior to the legislation being passed, detractors of the proposed smoke free law postulated that adults who had previously smoked in public places would instead be smoking in their homes. A displacement of their smoking activity from one arena to another could mean an increase in environmental tobacco smoke in locations where children are more likely to be present [38]. This would then have unintended consequences for the health of children and young people. Fortunately, HSE data showed that this was not the case, with children aged 4–15 significantly more likely to have undetectable cotinine in 2008 compared with 2006 [39].

Other ways in which HSE data have been used in policy evaluation and review are shown in Additional file 1: Table S1. Hypertension data have been used in several ways for policy evaluation and review, includes reviewing guidelines for the treatment of hypertension, the contribution of various health policies to CVD mortality in England (for which diabetes and cholesterol data were also used); and in examination of the association between primary care doctor numbers and the presence of undiagnosed cardiovascular disease. Cholesterol data were also used when examining the effects of the introduction of pay-by-performance for primary care and in reviewing the effects of allowing statins to be dispensed over-the-counter (without a prescription from a doctor). Examination of older people’s walking speeds had implications for the Mayor of London’s traffic calming strategy- in which the time given for pedestrians to cross the road would be reduced.

## Discussion

This paper presents specific examples of how the HSE examination data have been used by policy-makers. This includes identifying an issue amenable to policy-intervention, for example highlighting the greater prevalence of CKD overall than assumed from healthcare data. Measuring this problem raised the profile of CKD, allowing the context to be set for an increased drive for diagnosis and preventative action. HSE data have also been used in strategy development, with COPD prevalence data feeding in to development and implementation of government strategy and modelling for clinical guidance. HSE has been used in setting and monitoring targets, as for the 1992 *Health of the Nation* blood pressure targets; and in evaluation and review of health policy, for example examining the effects of the smoking ban on passive smoking by adults and children.

Some of the results cited in policy documents were from the official HSE reports, however there were also citations of peer-reviewed papers of secondary analyses of HSE data published in academic journals. Some policy documents used in-house analysis of the HSE data.

Previous studies examining the accuracy of self-reported health in comparison with health examination data have shown that for many health outcomes, self-reported results are inaccurate [10-13]. For example self-reported hypertension tends to be underestimated, so results from examination in a population survey may give more than twice the prevalence of hypertension than that found from an interview survey. Although comparison of self-reported diagnosis with medical records shows that the former is reasonably accurate, disease prevalence is generally under-reported compared with health examination studies, due to under-diagnosis. This is particularly true for hypertension and CKD e.g: [11,12]. Hypercholesterolaemia, diabetes, osteoarthritis and coronary heart disease are also underestimated by self-report, [13], as is obesity prevalence [10].

It was not possible to review all health policy documents for this study. In addition, policy making is a complex process and not all the sources that have fed into decision making are formally documented in final published documents. In interviews held as part of this work, a senior member of staff from the Department of Health said that often the influence of the HSE on pieces of policy work are indirect and unacknowledged. For example, it may be that a minister believes something which the data shows to be false. In this instance, a minister may accept the findings and adjust what they say from that point on, without any formal or public reference to what has affected their new position. That interviewee, and others, told OO that data can be absorbed into the consciousness and contribute to the thinking in the Department of Health without leading directly to a policy. Thus this paper has underestimated the ways in which the HSE has influenced health policy in the UK, with some instances not identified.

There are measurements which appear to have been used (at least explicitly) to a greater degree than others. Blood pressure and cotinine measurements were most frequently cited in the policy documents identified. There are also measurements that have been taken as part of the HSE for which we were unable to identify policy uses so far. These are measured C-reactive protein, fibrinogen, ‘flu antibodies, and gamma gt from blood; measured melatonin from the urine sample; and ECG data. Some of these measurements are very new and may not yet have had a chance to affect published policy documents. However, there was one example found of a missed opportunity, where ECG data may have been valuable but had not been located [40]. There were other examples of non-use, for example in the Standing Advisory Committee on Nutrition (SACN) 2010 *Iron and Health* report, which stated that HSE did not have specific data on iron deficiency anaemia, when in fact this had been collected. Qualitative work with Department of Health staff has provided some reasons for under-use of HSE data.

## Conclusions

There is much anecdotal evidence that health examination surveys are valued by policy makers [2]. When the UK Secretary of State for Health was asked in Parliament what benefits have resulted from funding and carrying out the HSE, the response was that HSE is a major vehicle, providing valuable annual data about the nation's health and the information is used to underpin strategies for promoting better health [41]. However, until now there has not be documented evidence of specific examples in which policy was shaped by collection of health examination data through the HSE. This paper shows the ways in which a health examination survey can contribute to national health policy, demonstrating that is it a useful part of a national health information system.

## Abbreviations

CKD: Chronic kidney disease; COPD: Chronic obstructive pulmonary disease; GP: General practitioner (Primary care practitioner); HSE: Health survey for England; QMAS: Quality management analysis system.

## Competing interests

JM is funded by the HSCIC to work on the HSI series. Both the HSCIC and the Department of Health fund the HSE. This study was unfunded and the HSE funders were not involved in the decision to undertaken this work nor to publish it. OO has no conflicts of interest to declare.

## Authors’ contributions

JM conceived of the study. OO interviewed members of the Department of Health and the Health and SCIC and reviewed the literature to identify examples of use of the HSE. Both authors contributed to drafting the final manuscript. Both authors read and approved the final manuscript.

## Supplementary Material

Additional file 1: Table S1 Specific examples of HSE examination data used in policy making and monitoring. Click here for file
