# Supplementary material for: A review of the use of health examination data from the Health Survey for England in government policy development and implementation
Source: Arch Public Health. 2014 Jul 15;72(1):24. doi: 10.1186/2049-3258-72-24 (PMC4128608; doi:10.1186/2049-3258-72-24)
Supplement: Additional file 1: Table S1 — Specific examples of HSE examination data used in policy making and monitoring. [file 2049-3258-72-24-S1.doc]

**Table S2: Specific examples of Health Survey for England examination data used in policy making and monitoring**

|  | **Measurements** | **Recognition of health issue** | **Strategy Development** | **Target Setting and Monitoring** | **Evaluation and Review** |
| --- | --- | --- | --- | --- | --- |
| **Hypertension** | **Blood pressure** | Reports blood pressure by social class to demonstrate inequality. *Acheson 1998*  Reports prevalence of measured hypertension and compares with prevalence of doctor diagnosed and treated hypertension. *DH 1999*  Reports prevalence data. Highlights hypertension as a preventable cause of morbidity and mortality. *CMO 2002*  Presents prevalence of hypertension by income quintile, highlighting inequality. *DH 2009*  Presents prevalence of hypertension by age and sex. *CMO 2012*  Prevalence of hypertension by income is discussed. *Marmot 2010*  Used in modelling the economic impact of CKD. *Kerr 2012* [19] | Used in economic modelling for vascular checks. *DH 2008* | Setting and monitoring the Health of the Nation Target. *DH 1992* [28]  Monitoring the prevalence of hypertension. *CMO 2004* and *CMO 2005*  Monitoring the hypertension indicator from the World Health Organization (WHO) Commission on Social Determinants of Health. *DH 2009*  Monitoring prevalence of hypertension. *Falaschetti et al. 2009* [32] | Reviews guidelines on treatment for hypertension. [*Primatesta et al. 2001*](../Hypertension.)  [Reviews the contribution of various health policies to CVD mortality in England using prevalence data. *Bajekal et al. 2012*](../Hypertension.)  Uses hypertension prevalence data in a model examining undiagnosed cardiovascular disease and the numbers of primary care doctors. *Soljak et al. 2011.* |
| **Cholesterol** | **Serum total & HDL cholesterol;**  **Fasting serum LDL cholesterol & triglycerides** | Presents prevalence of raised cholesterol by age and sex. *CMO 2012*  Example of a parliamentary question answered using HSE data. *Hansard 2006a* | Used in economic modelling for vascular checks. *DH 2008* |  | Evaluated the introduction of “pay-by-performance” in primary care on lipid-levels. *Mindell 2011*  Evaluated the effects of allowing statins to be provided over the counter on cholesterol levels. *Mainous et al. 2010*  Reviews the contribution of various health policies to CVD mortality in England using prevalence data. *Bajekal et al. 2012* |
| **Physical function** | **Grip strength;**  **Physical function, balance;**  **Walking Speed** |  |  |  | Demonstrated that most older people would be unable to cross the road within the time allotted under the Mayor of London’s plans to smooth traffic flow. *Asher et al. 2012* |
| **Physical activity, & cardiovascular fitness** | **Accelerometry**  **Step test** | Accelerometry results presented, highlighting the prevalence of physical inactivity. *CMO 2010*  Accelerometry data used to show prevalence of physical inactivity. *DfT 2010*  Prevalence of physical inactivity by income is reported. *Marmot 2010*  Accelerometry data used to show prevalence of physical inactivity. *DH 2011c* |  |  |  |
| **Passive Smoking** | **Serum cotinine; Salivary cotinine** | Demonstrates inequality in the burden of smoking by social class using measured cotinine. *Acheson 1998*  Demonstrates high cotinine in non-smoking spouses of smokers. *Jarvis et al. 2001*  Uses cotinine to examine secondhand smoke. *SCOTH 2004*  *Health Select Committee 2006 [36]* references *Tobacco Advisory Group of the Royal College of Physicians 2005,* which presents data on cotinine by age, sex, income and more.  Discusses data from HSE such as decrease in children’s exposure to smoke over the previous years. *DH 2011b*  Presents evidence on passive smoking by children. *Tobacco Advisory Group of the Royal College of Physicians 2010.* |  | Monitoring the third objective of the strategy (protecting families and communities) by testing cotinine levels in children to validate the proportion of homes  where both parents are smokers but where the home itself has been declared ‘smokefree’. *HM Government 2010* | Evaluation of the smoking ban to be done using HSE cotinine data*. Select Committee on Health 2005*  Examines the prevalence of smoke-free homes. *Jarvis et al. 2009*  Uses many academic papers based on HSE to evaluate previous government policies on smoking. *HM Government 2010*  Evaluation of the smoking ban. Evidence review for the DH *Bauld 2011* [34]; academic papers *Sims et al. 2012* [35]; *Jarvis et al. 2012* [37]. |
| **Kidney Function** | **Urine sample – albumin** | Models prevalence of CKD across England. *Kearns 2009*  Examines the association between urinary albumin and cognitive function. *Llewellyn et al. 2010*  Highlights undiagnosed CKD as a problem in England using prevalence data. *O’Donoghue 2010*  Presents prevalence of chronic kidney disease. *CMO 2012*  Used in modelling the economic impact of Chronic Kidney Disease *Kerr 2012 a,b* [19,20] | Used in modelling the economic impact of Chronic Kidney Disease *Kerr, 2012a ,b* [19,20] |  |  |
| **Lung Function** | **Lung function** | *Shahab et al. 2006* [22]and *Nacul et al. 2007 [*23] used prevalence data to examine measured prevalence of COPD compared with doctor diagnoses. These were used in *DH 2011d* and *British Lung Foundation 2007* [25].  Presents lung function by social class to demonstrate inequality. *Acheson 1998*  Models undiagnosed COPD prevalence. *Walford et al. 2011* | Used in modelling to compare COPD treatments. *NICE 2010*  Prevalence data were used with clinicians to make the case for the COPD outcomes strategy *DH 2011d*  COPD prevalence data were used in the equality impact assessment of the COPD outcomes strategy. *DH 2011e* |  |  |
| **Diabetes** | **Blood glycated haemoglobin; Fasting blood glucose** | Presents prevalence of undiagnosed diabetes. *British Heart Foundation 2006*  Example of a parliamentary question answered using HSE data. *Hansard 2006b*  Prevalence of diabetes by income is discussed. *Marmot 2010*  Prevalence of diagnosed and undiagnosed diabetes are modelled for England. *Holman et al. 2011* | Used in economic modelling for vascular checks. *DH 2008* |  | Reviews the contribution of various health policies to CVD mortality in England using prevalence data. *Bajekal et al. 2012* |
| **Anaemia** | **Haemoglobin, Mean Corpuscular Volume (MCV), ferritin, serum transferrin, vitamin B12** | Example of a parliamentary question answered using HSE data. *Hansard 2003b*  Prevalence of anaemia by age, gender and ethnicity. *SACN 2010* |  |  |  |
|  | **Blood sample – Immuno-globulin-E/House Dust Mite Immuno-globulin-E** | Prevalence and risk factors for raised dust-mite specific IgE. *Court et al. 2002* |  |  |  |
|  | **Blood sample – vitamin D** | Prevalence of hypovitaminosis D presented. *Jackson 2007*  Explores the association between hypovitaminosis D and cognition. *Llewellen et al 2009.*  Prevalence and risk factors for hypovitaminosis D. Recommends policy action. *Hirani et al. 2010* |  |  |  |

**References**

Acheson D (1998) Independent Inquiry into Inequalities in Health in England. TSO, London

Asher L, Aresu M, Faslaschetti E, Mindell JS (2012). Most older pedestrians are unable to cross the road in time a cross sectional study. Age Aging 41:690-694.

Bajekal M, Scholes S, Love H, Hawkins N, O’Flaherty M, Raine R, Capewell S (2012) Analysing recent socioeconomic trends in coronary heart disease mortality in England 2000-2007: a population modelling study. Plos Med 9:e1001237.

British Heart Foundation (2006) Active for Later Life. BHF National Centre, Loughborough

CMO (2001) On the state of the public health: Annual Report of the Chief Medical Officer 2001. Department of Health, London <http://webarchive.nationalarchives.gov.uk/20080906005344/http://dh.gov.uk/en/Aboutus/MinistersandDepartmentLeaders/ChiefMedicalOfficer/CMOPublications/CMOAnnualReports/index.htm> Accessed 23 Jul 2013

CMO (2004) On the state of the public health: Annual Report of the Chief Medical Officer 2003. Department of Health, London <http://webarchive.nationalarchives.gov.uk/20080906005344/http://dh.gov.uk/en/Aboutus/MinistersandDepartmentLeaders/ChiefMedicalOfficer/CMOPublications/CMOAnnualReports/index.htm> Accessed 23 Jul 2013

CMO (2005) On the state of the public health: Annual Report of the Chief Medical Officer 2004. Department of Health, London <http://webarchive.nationalarchives.gov.uk/20080906005344/http://dh.gov.uk/en/Aboutus/MinistersandDepartmentLeaders/ChiefMedicalOfficer/CMOPublications/CMOAnnualReports/index.htm> Accessed 23 Jul 2013

CMO (Chief Medical Officer) (2010). On the state of public health: Annual report of the Chief Medical Officer 2009. Department of Health, London

CMO (Chief Medical Officer) (2012). Annual Report of the Chief Medical Officer 2012. Department of Health, London

Court CS, Cook DG, Strachan DP (2002). Comparative epidemiology of atopic and non-atopic wheeze and diagnosed asthma in a national sample of English adults. Thorax 57:951-957.

Dept for Transport (2010) Active Travel Strategy. Department for Transport/Department of Health, London

Department of Health (1999) White Paper Saving lives: Our healthier nation. Department of Health, London

Department of Health (2008) Economic modelling of vascular checks. DH, London [www.healthcheck.nhs.uk/national_guidance/department_of_health_publications/](http://www.healthcheck.nhs.uk/national_guidance/department_of_health_publications/) Accessed 18 July 2013

Department of Health (2009) Tackling Health Inequalities: 10 Years On A review of developments in tackling health inequalities in England over the last 10 years. London, DH.

Department of Health (2011c). Start active, stay active: a report on physical activity from the four home countries' Chief Medical Officers. Department of Health, Physical Activity, Health Improvement and Protection, London

Department of Health (2011d) The Outcomes Strategy for Chronic Obstructive Pulmonary Disease (COPD) and Asthma. DH, London <https://www.gov.uk/government/publications/an-outcomes-strategy-for-people-with-chronic-obstructive-pulmonary-disease-copd-and-asthma-in-england> Accessed 19^th^ July 2013

Department of Health (2011e). An outcomes strategy for chronic obstructive pulmonary disease (COPD) and asthma. Assessment of the Impact on Equality. DH, London <https://www.gov.uk/government/uploads/system/uploads/attachment_data/file/216140/dh_128427.pdf> Accessed 19^th^ July 2013

Hansard (2003b) Parliamentary Questions. House of Commons Hansard Written Answers for 8 September 2003 PQ 127538 Anaemia <http://www.publications.parliament.uk/pa/cm200203/cmhansrd/vo030908/text/30908w41.htm> Accessed 24^th^ Jul 2013

Hansard (2006a). Parliamentary Questions. House of Commons Hansard Written Answers for 24 April 2006 PQ 64112 Cholesterol levels. <http://www.publications.parliament.uk/pa/cm200506/cmhansrd/vo060424/text/60424w40.htm> Accessed 24th Jul 2013

Hansard (2006b) Parliamentary Questions. House of Commons Hansard Written Answers for 5 June 2006 [PQ 71084]. Diabetes. <http://www.publications.parliament.uk/pa/cm200506/cmhansrd/vo060605/text/60605w0070.htm> Accessed 24th Jul 2013

Hirani V, Tull K, Ali A, Mindell J (2010) Urgent action needed to improve vitamin D status among older people in England Age Aging. 39:62-8.

HM Government (2010) A smoke free future: A New Vision for Tobacco Control in England Department of Health , London <http://webarchive.nationalarchives.gov.uk/20100509080731/http://dh.gov.uk/en/Publicationsandstatistics/Publications/PublicationsPolicyAndGuidance/DH_111749> Accessed 19^th^ July 2013

Holman N, Forouhi NG, Goyder E, Wild SH (2011) The Association of Public Health Observatories (APHO) Diabetes Prevalence Model estimates of total diabetes prevalence for England, 2010-2030. Diabetes Med 28:575-582.

Jackson AA (2007) Update on Vitamin D Position Statement by the Scientific Advisory Committee on Nutrition. TSO The Stationery Office, London

Jarvis MJ, Feyerabend C, Bryant A, Hedges B, Primatesta P (2001) Passive smoking in the home plasma cotinine levels in non-smokers with smoking partners. Tob Control 10:368-374

Jarvis MJ, Mindell J, Gilmore A, Feyerabend C, West R (2009) Smoke-free homes in England prevalence, trends and validation by cotinine in children. Cross-sectional survey data. Tob Control 18:491-495.

Kearns B (2009) Chronic Kidney Disease Prevalence Modelling. Briefing Document. EMPHO, Birmingham

Llewellyn DJ, Langa KM, Lang IA (2009). Serum 25-hydroxyvitamin D concentration and cognitive impairment. Journal of geriatric psychiatry and neurology 22:188-95.

Llewellyn DJ, Lang IA, Langa KM, Muniz-Terrera G, Phillips CL, Cherubini A, Ferrucci L, Melzer D (2010). Vitamin D and risk of cognitive decline in elderly persons. Arch Intern Med 170:1135-1141.

Mainous AG 3^rd^, Baker R, Everett CJ, King DE (2010) Impact of a policy allowing over-the-counter statins. Qual Primary Care. 18:301-306

Marmot M (Chair) (2010). Fair Society, Healthy Lives. The Marmot Review, London

Mindell J, Aresu M, Zaninotto P, Falaschetti E, Poulter N (2011) Improving lipid profiles and increasing use of lipid-lowering therapy in England results from a national cross-sectional survey 2006 Clin Endocrinol 75:621-627

O’Donoghue D (2010) Renal Tsar’s Blog. Health Survey England confirms CKD is common and shines a light on proteinuria <http://renaltsar.blogspot.com/2010/12/health-survey-england-confirms-ckd-is.html> Accessed 23 July 2013

Primatesta P, Brookes M, Poulter NR (2001) Improved hypertension management and control: results from the health survey for England 1998. Hypertension 38:827-832.

SACN (Scientific Advisory Committee on Nutrition) (2010). Iron and Health report. TSO, London

SCOTH (Scientific Committee on Tobacco and Health) (2004). Secondhand Smoke Review of evidence since 1998 Update of evidence on health effects of secondhand smoke. Department of Health, London

Select Committee on Health (2005). First report. [www.publications.parliament.uk/pa/cm200506/cmselect/cmhealth/485/48514.htm](http://www.publications.parliament.uk/pa/cm200506/cmselect/cmhealth/485/48514.htm) Accessed 19^th^ July 2013

[Soljak M](http://www.ncbi.nlm.nih.gov/pubmed?term=Soljak M%5BAuthor%5D&cauthor=true&cauthor_uid=21414221), [Samarasundera E](http://www.ncbi.nlm.nih.gov/pubmed?term=Samarasundera E%5BAuthor%5D&cauthor=true&cauthor_uid=21414221), [Indulkar T](http://www.ncbi.nlm.nih.gov/pubmed?term=Indulkar T%5BAuthor%5D&cauthor=true&cauthor_uid=21414221), [Walford H](http://www.ncbi.nlm.nih.gov/pubmed?term=Walford H%5BAuthor%5D&cauthor=true&cauthor_uid=21414221), [Majeed A](http://www.ncbi.nlm.nih.gov/pubmed?term=Majeed A%5BAuthor%5D&cauthor=true&cauthor_uid=21414221) (2011). Variations in cardiovascular disease under-diagnosis in England: national cross-sectional spatial analysis. [BMC Cardiovasc Disord.](http://www.ncbi.nlm.nih.gov/pubmed/21414221) 17;11:12.

Tobacco Advisory Group of the Royal College of Physicians (2010). Passive smoking and children. Royal college of Physicians, London.

Tobacco Advisory Group of the Royal College of Physicians (2005). The medical case for clean air in the home, at work and in public places. Royal college of Physicians, London.

Walford H, Ramsay L, Soljak M, Majeed A (2011) COPD Prevalence Modelling Briefing Document. ERPHO, Cambridge
